# Effect of Histopathological Findings of Gastric Specimens Resected During Laparoscopic Sleeve Gastrectomy on Weight Loss Success: A Retrospective Analysis of 599 Patients

**DOI:** 10.7759/cureus.60881

**Published:** 2024-05-22

**Authors:** Muhammed Taha Demirpolat, Mehmet Muzaffer İslam, Emine Maksude Ceylan, Furkan Aykıt, Mustafa Satır, Irem Güvendir Bakkaloglu, Mehmet Erman Bacaksız, Metin Yücel, Abdullah Sisik

**Affiliations:** 1 General Surgery, University of Health Sciences, Umraniye Training and Research Hospital, Istanbul, TUR; 2 Emergency Medicine, University of Health Sciences, Umraniye Training and Research Hospital, Istanbul, TUR; 3 Pathology, University of Health Sciences, Umraniye Training and Research Hospital, Istanbul, TUR; 4 General Surgery, Dr. HE Obesity Clinic, Istanbul, TUR

**Keywords:** malignancy risk, total weight loss, weight loss success, histopathological findings, laparoscopic sleeve gastrectomy

## Abstract

Background: Even though there aren't enough studies on long-term outcomes, laparoscopic sleeve gastrectomy (LSG) is the most common procedure among weight loss surgeries. In this study, we aimed to evaluate the histopathological results of resected stomach specimens of patients who underwent LSG and to analyze the effect of histopathological results on weight loss success.

Methods: The patients were divided into two groups according to histopathological results of the pathology specimens: abnormal (chronic active gastritis, chronic inactive gastritis, neoplasias) and normal. If the excess weight loss percentage (EWL%) values were over 70% at the end of the first year following LSG, the patients were considered successful in terms of weight loss. The groups were compared in terms of age, gender, preoperative body mass index (BMI) value, as well as postoperative first-year BMI, EWL%, total weight loss percentage (TWL%), and successful patient percentage.

Results: A total of 599 patients were included in this study. When the patients were dichotomized according to their pathology results as normal or abnormal, 101 (%83.5) of the patients with normal pathology results had EWL% greater than 70%. On the contrary, 356 (74.5%) of the patients with abnormal pathology results had EWL% greater than 70%, and this difference was statistically significant (p=0.038).

Conclusion: Patients with normal histopathologic examination results of resected gastric specimens after LSG are more successful than the patient population with abnormal histopathologic results in terms of the percentage of patients with EWL% above 70 at the end of the first postoperative year. We recommend routine histopathologic analysis of gastric specimens after LSG in severely obese patients.

## Introduction

Obesity, defined as the accumulation of excess fat in the body, is a serious public health problem that leads to many life-threatening comorbid diseases, and its prevalence is increasing each day [1,2]. Although many methods have been developed to deal with obesity, surgery remains the best approach to achieving sustainable weight loss [3,4]. Even though there aren't enough studies on long-term outcomes, laparoscopic sleeve gastrectomy (LSG) is the most common procedure among weight loss surgeries. LSG is a procedure based on the resection of the lateral 70-80% of the stomach, including partial antrum and complete fundus. This method provides weight loss both by limiting the amount of food consumed and by suppressing the secretion of ghrelin hormone [5,6].

There is no strong evidence to prove the necessity of histopathologic examination of resected gastric specimens during LSG, because, unlike oncologic surgeries, the purpose of resection here is only to reduce gastric volume. Therefore, this decision is up to the initiative of the institutions [7,8]. While some researchers have stated that there are more abnormal histopathological results in obese individuals and therefore specimens should be followed carefully [9,10], some researchers have suggested that there is no significant difference from normal society, so histopathological examination is not necessary [11,12].

There are no sufficient reports in the literature evaluating the histologic results of pathology specimens after LSG and investigating whether there is a relationship between the weight loss success of the patients and the histologic findings of the specimens.

In this study, we aimed to evaluate the necessity of histopathologic analysis of resected gastric specimens and the effect of histopathologic results on weight loss success in patients who underwent LSG.

## Materials and methods

Study design and population

Patients who underwent LSG due to morbid obesity at the general surgery clinic of the Umraniye Training and Research Hospital between January 2018 and June 2022 were analyzed retrospectively. The study included patients whose body mass index (BMI) was 35 or higher and who underwent LSG. Patients who underwent other bariatric surgical procedures, had been actively smoking, had consumed alcohol, and had a lack of data were excluded from the study. Informed consent was obtained from all patients for the study. This study was performed in line with the principles of the Declaration of Helsinki. Approval for the study was received from the Ethics Committee of Umraniye Training and Research Hospital (approval number: 06.10.2023/226333876). 

Data collection

The demographic data of the patients and the histopathological features of the resected stomach specimens were recorded. The weight, BMI, total weight loss percentage (TWL%), and excess weight loss percentage (EWL%) values of the patients at the end of the first year and preoperatively were recorded. Body weight (kg)/height (m^2^) was the formula used for calculating BMI. The optimal BMI of 25 kg/m^2^ was acknowledged, and the patients' ideal weight was determined using 25×height^2^ (m^2^). TWL%=((initial weight (kg)−first-year weight))×100/initial weight (kg) was calculated with the formula. EWL%=(initial weight (kg)−first-year weight (kg)/initial weight (kg)−ideal weight)×100 was calculated with the formula [13].

All patients were evaluated with preoperative upper gastrointestinal endoscopy (UGIE), but routine biopsy was not performed during preoperative UGIE. Biopsy and histopathological examination were performed only on patients suspected of any malignant or pre-malignant lesion. The timing of preoperative UGIE was in the last one month prior to surgery. Following the surgery, resected stomach samples were sent to the pathology department in plastic containers containing formaldehyde. Specimens were evaluated by gastrointestinal pathologists. Histopathological findings were classified according to the Sydney classification [14].

The patients were divided into two groups according to histopathological results of the pathology specimens: abnormal (gastritis ulcer, duodenal ulcer, chronic active gastritis (CAG), chronic inactive gastritis (CIG), neoplasias) and normal (without any gastritis and neoplasia). If the EWL% values were over 70% at the end of the first year following LSG, the patients were considered successful in terms of weight loss [15]. The groups were compared in terms of age, gender, preoperative BMI value, as well as postoperative first-year BMI, EWL%, TWL%, and successful patient percentage. We also grouped the patients according to the features of benign abnormal histopathologic results (presence of inflammation, atrophy, intestinal metaplasia, *Helicobacter pylori*, and lymphoid follicle) and compared these results with the weight-related parameters. Receiver operating characteristic (ROC) curve analysis was applied for the estimated cut-off age for the detection of neoplasia. Patients were also divided into two groups according to age: the age group below the cut-off value (younger patients) and the age group above (older patients). Younger and older patient groups were also compared in terms of histopathological findings.

Statistical analysis

IBM SPSS Statistics for Windows, Version 29.0 (Released 2023; IBM Corp., Armonk, New York, United States) was used for statistical analysis of the collected data. Whether continuous data conformed to normal distribution was analyzed with the Shapiro-Wilk test. Since all continuous data were abnormally distributed, they were expressed as median (25-75% quartile), and the Mann-Whitney U test was used for comparisons between groups. Categorical data were expressed as frequency (%), and the chi-squared test was used for comparisons between groups. ROC analysis was performed to determine the optimal threshold value for age, and the threshold value was determined with the Youden index. Statistical significance level was determined as p<0.05. For the multilogistic regression analysis, binary regression forced-entry method was used. The goodness of fit was tested with the Hosmer-Lemeshow test, and the model performance was evaluated with Nagelkerke R2 and overall accuracy.

## Results

A total of 599 patients were included in this study. The median age and BMI of the participants were 36 (28-44) and 44 (42-47) kg/m^2^, respectively, and 493 (82.3%) of them were female. The median EWL% was 85.8% (71.3-99.4), and 457 (76.3%) of all the patients had >70% EWL one year after the LSG. Regarding the results of the pathological examination, 121 (20.2%) patients had normal histopathologic results, while 478 (79.8%) patients had abnormal histopathologic results. 

A total of nine (1.5%) patients were diagnosed with neoplasia, of whom three (0.5%) were gastrointestinal stromal tumors (GIST) and six (1%) were neuroendocrine tumors (NET). Preoperative UGIE and biopsy allowed for the histological diagnosis of three out of nine neoplasia patients. Two of these patients had GIST diagnoses and one had NET diagnoses. The timing of surgery was not changed because the lesions in these patients were in the regions that were supposed to be resected during the LSG. The basic characteristics of the total population are summarized in Table 1.

**Table 1:** Basic characteristics of the total population BMI: body mass index; TWL: total weight loss; EWL: excess weight loss; NET: neuroendocrine tumor; GIST: gastrointestinal stromal tumor

	Median (25-75% quartiles) or n (%)
Age (in years)	36 (28-44)
Sex (female)	493 (82.3%)
Preoperative weight (kg)	118 (109-130)
Preoperative BMI kg/m^2^	44 (42-47)
Postoperative weight (kg)	74 (67-84)
TWL%	37.9 (31.9-42.9)
EWL%	85.8 (71.3-99.4)
EWL% >70%	457 (76.3%)
Pathology results	
Normal	121 (20.2%)
Chronic inactive gastritis	222 (37.1%)
Active gastritis	247 (41.2%)
Neoplasia	9 (1.5%)
NET	6 (66.7%)
GIST	3 (33.3%)
Dichotomized pathology results	
Normal	121 (20.2%)
Abnormal	478 (79.8%)

When the patients were dichotomized according to their pathology results as normal or abnormal, 101 (83.5%) of the patients with normal pathology results had EWL% greater than 70%. On the contrary, 356 (74.5%) of the patients with abnormal pathology results had EWL% greater than 70%, and this difference was statistically significant (p=0.038). It was determined that the patients in the normal group were significantly better in terms of weight at the end of the first year after LSG compared to those in the abnormal group (p=0.006). The comparison of the basic characteristics between the patients with normal and abnormal pathology results is summarized in Table 2. 

**Table 2:** The comparison of the basic characteristics between the patients with normal and pathological pathology results TWL: total weight loss; EWL: excess weight loss; BMI: body mass index

	Normal group	Abnormal group	P-value
EWL >70%, n (%)	101 (83.5%)	356 (74.5%)	0.038
EWL (%), median (25-75% quartiles)	87.3 (76-102.6)	85.5 (69.7-99.1)	0.092
TWL (%), median (25-75% quartiles)	38.4 (33.9-43.7)	37.7 (31.2-42.6)	0.096
Age, median (25-75% quartiles)	35 (27-44)	37 (29-44)	0.472
Sex (female), n (%)	103 (85.1%)	390 (81.6%)	0.363
Preoperative weight (kg), median (25-75% quartiles)	115 (108-125)	119 (110-130)	0.055
Preoperative BMI kg/m^2^, median (25-75% quartiles)	44 (42-48)	44 (42-47)	0.809
Postoperative weight (kg), median (25-75% quartiles)	71 (65-80)	75 (67-85)	0.006
Postoperative BMI kg/m^2^, median (25-75% quartiles)	27.8 (24.6-30.1)	27.9 (25.1-31.2)	0.095

**Figure 1:**
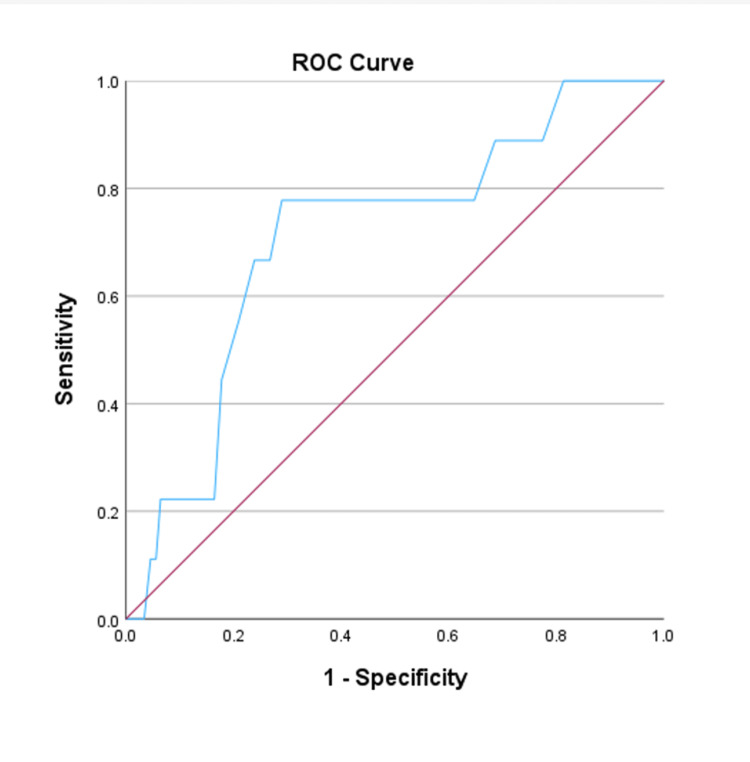
ROC curve analysis for the estimated cut-off age for the detection of neoplasia ROC: receiver operating characteristic

We analyzed the patients to provide an optimal age cut-off for the occurrence of neoplasia, ran an ROC analysis, and applied the Youden index (AUC=0.711 (95%CI=0.547-875), p=0.030) (Figure 1). 

Analysis of histopathologic results after LSG identified a cut-off age of 42.5 years for a significant increase in the incidence of neoplasia in patients with obesity. When the histopathological results were analyzed by age, the older patients were found to be significantly more neoplastic (p=0.004), but there was no significant difference in abnormal histopathological findings between the younger and older groups (p=0.816) (Table 3).

**Table 3:** The comparison of the variables between the age groups (cut-off=42.5 years)

	Younger patients	Older patients	P-value
Histopathological results			0.004
Non-neoplasia, n (%)	419 (99.5%)	171 (96.1%)	
Neoplasia, n (%)	2 (0.5%)	7 (3.9%)	
Histopathological results			0.816
Normal, n (%)	84 (20%)	37 (20.8%)	
Pathological, n (%)	337 (%80)	141 (%79.2)	

In logistic regression analysis, variables influencing weight loss success, based on the EWL 70% threshold value, were examined. The "normal" group and the "malignancy" group were excluded from the regression analysis due to the absence of pathological subgroupings. Since preoperative weight and BMI exhibited a high correlation, preoperative weight was excluded from the model. The assumption of goodness of fit was met (Hosmer-Lemeshow p=0.175). The model accounted for 19.3% of the variance and accurately predicted 78.3% of all cases (Nagelkerke R2=0.193). Age and preoperative BMI were determined as independent predictors of weight loss success according to the EWL 70% threshold value (p<0.001 and p<0.001, respectively). Histopathological subgroups did not significantly contribute to the model. Based on this analysis, each unit increase in age was associated with a 0.951 times decrease in weight loss success, while each unit increase in preoperative BMI value was linked to a 0.834 times decrease in weight loss success (Table 4).

**Table 4:** Regression analysis of factors influencing weight loss success based on the EWL 70% threshold value BMI: body mass index; IM: intestinal metaplasia; HP: *Helicobacter pylori*; CAG: chronic active gastritis Logit = 9.124 + x1*-0.05 + x2*-0.136 + x3*0.291 + x4*0.310 + x5*1.433 + x6*-0.519 + x7*0.022 + x8*-0.254

		B coefficient	Standard error	P-value	Odds ratio (95%CI)
x_1_	Age	-0.050	0.012	<0.001	0.951 (0.929-0.973)
x_2_	Preoperative BMI	-0.136	0.023	<0.001	0.834 (0.834-0.913)
x_3_	Sex (male)	0.291	0.315	0.355	1.338 (0.722-2.481)
x_4_	Inflammation (presence)	0.310	0.304	0.309	1.363 (0.750-2.475)
x_5_	Atrophy (presence)	1.433	0.843	0.089	4.191 (0.804-21.857)
x_6_	IM (presence)	-0.519	0.562	0.355	0.595 (0.198-1.789)
x_7_	HP (presence)	0.022	0.315	0.945	1.022 (0.551-1.894)
x_8_	CAG (presence)	-0.254	0.324	0.433	0.775 (0.411-1.463)
	Intercept	9.124	1.169	<0.001	NA

There were no significant differences between the inflammation, atrophy, intestinal metaplasia, *Helicobacter pylori*, and lymphoid follicle groups in terms of preoperative weight, preoperative BMI, postoperative weight, postoperative BMI, EWL%, and TWL%. The comparison between benign abnormal histopathological findings, its subgroups, and weight loss success is summarized in Table 5.

**Table 5:** Association between abnormal histopathologic findings, its subgroups, and weight loss IM: intestinal metaplasia; HP: *Helicobacter pylori*; LF: lymphoid follicle; CIG: chronic inactive gastritis; CAG: chronic active gastritis

	Preop weight median (25-75% quartiles)	Preop BMI median (25-75% quartiles)	12th-month EWL% median (25-75% quartiles)	12th-month TWL% median (25-75% quartiles)
CIG (N=222)	120 (110-130)	44 (42-48)	87 (71-102)	38 (33-44)
CAG (N=247)	118 (109-130)	44 (42-47)	84 (70-97)	38 (31-42)
P-value	0.985	0.769	0.153	0.068
Mild inflammation (N=222)	120 (109-130)	44 (41-48)	87 (70-99)	38 (32-43)
Moderate-severe inflammation (N=247)	118 (110-130)	44 (42-47)	85 (70-99)	39 (31-42)
P-value	0.689	0.445	0.536	0.754
Atrophy (-) (N=451)	118 (110-130)	44 (42-47)	86 (70-99)	38 (31-43)
Atrophy (+) (N=18)	119 (110-123)	45 (42-46)	87 (73-100)	40 (33-43)
P-value	0.749	0.718	0.855	0.742
IM (-) (N=434)	119 (110-130)	44 (42-47)	86 (70-99)	38 (32-43)
IM (+) (N=35)	117 (107-123)	44 (41-46)	83 (68-98)	36 (28-43)
P-value	0.113	0.439	0.461	0.330
HP (-) (N=193)	120 (110-129)	44 (42-48)	87 (70-102)	38 (32-43)
HP (+) (N=276)	118 (109-130)	44 (42-47)	85 (70-97)	38 (31-42)
P-value	0.979	0.583	0.201	0.231
LF (-) (N=77)	116 (107-130)	43 (41-46)	89 (68-100)	37 (30-43)
LF (+) (N=392)	119 (110-130)	44 (42-47)	85 (70-99)	38 (31-42)
P-value	0.310	0.183	0.487	0.924

## Discussion

This study contributes to the literature by examining the role on weight loss of postoperative histopathologic examination of the resected gastric specimens during LSG. 

Furthermore, one of the most effective factors in conducting this study was the disagreement on the necessity for routine histopathological examination of gastric specimens resected during LSG, despite the fact that multiple studies have demonstrated a relationship between obesity and an increased risk of malignancy [16-19].

In our study, it was found that the patient with normal histopathological results of the specimens following LSG was significantly more successful than the patient with abnormal histopathologic findings in terms of the percentage of patients with EWL% >70% at the end of the first year, but no significant difference was found between the groups in terms of EWL% and TWL%. The homogeneity of the groups is demonstrated by the similar preoperative median BMI values of the patients. The number of patients with abnormal histopathological findings is 478 (79.8%) and is compatible with the literature findings [20]. Another important result is that while the number of patients with neoplasia was significantly higher in the older group, no significant difference was found between the younger and older groups in terms of other abnormal histopathological findings.

Erkinuresin et al. [16] found significantly higher 12th-month EWL% in patients without intestinal metaplasia and with inactive gastritis compared to patients with active gastritis. Tomasicchio et al. [21] compared the 12th-month EWL% of patients with chronic gastritis and patients without chronic gastritis and found that the EWL% of patients without chronic gastritis was higher but did not reveal a significant difference. In our study, histopathological analysis of the specimens revealed no significant difference in weight loss between the abnormal and normal patient groups in terms of EWL% and TWL%, while we found that the EWL% >70% patient percentage was significantly higher in the normal group. Although there is no significant difference in the EWL%, there is a significant difference in the percentage of successful patients, suggesting that having normal histopathologic findings positively correlates with weight loss success. This result can be linked to a hormonal feedback mechanism. In addition, in LSG patients whose gastritis was identified during the preoperative period, performing surgery following medical treatment for gastritis can increase the percentage of successful patients. Unlike the study by Erkinuresin et al. [16] who compared groups of patients with abnormal histopathologic findings, our study compared patient populations with normal and abnormal histopathologic findings, which may explain this different result.

Alessandris et al. [22] examined gastritis specimens of 501 LSG patients and found 12 (2.4%) neoplasms. Keshishian et al. [23] detected carcinoid tumors in three (1.5%) of 198 patients and found a high incidence of carcinoids in patients with obesity compared to the general population. This result sheds doubt on the influence of obesity in the pathogenesis of tumors. In our study, neoplasia was detected in nine (1.5%) patients with obesity. In the literature, the high incidence of neoplasia in patients with obesity has been attributed to the metabolic and hormonal effects of obesity [24]. Unhealthy eating habits may also contribute to the higher incidence of neoplasia in patients with obesity by increasing the risk of cellular transformation in the gastrointestinal tract. Further studies are required to explain this high incidence of neoplasia in patients with obesity. We also believe that histopathologic analysis of resected specimens should be routinely performed in LSG patients.

Strengths and limitations

We believe that this study will contribute to the literature in terms of two aspects. The first of these is that the relationship between histopathologic results of the specimens and weight loss success is a topic that has not been sufficiently studied in the bariatric literature. To the best of our knowledge, there is a paucity of studies in the literature regarding this issue and the findings of some of these studies. The discrepancy in this topic has also revealed that further studies are required to assert that weight loss following LSG and histopathological findings are precisely associated with each other. The second noteworthy finding of our study is that it contributed to the resolution of the uncertainty about whether histopathologic analysis of specimens resected during LSG is required. IM and atrophy were found in 18 (3%) patients in our study. Histopathologic analysis of the specimens after LSG will allow close follow-up of these patients by determining the results of IM and atrophy, which are two predisposing factors of gastric adenocarcinoma. The important limitations of this study are that it was a retrospective and a single-center study. At the same time, the fact that parameters affecting abnormal histopathologic findings such as frequent use of NSAIDs, presence of diabetes mellitus, and use of several medications were not included in the study can also be considered among the limitations. Prospective and multicenter studies, the examination of different patient groups, and the evaluation of long-term outcomes are needed for future studies.

## Conclusions

Patients with normal histopathologic examination results of resected gastric specimens after LSG are more successful than the patient population with abnormal histopathologic results in terms of the percentage of patients with EWL% above 70 at the end of the first year. When patients with benign abnormal histopathology are analyzed, having CAG or CIG does not affect weight loss success. We recommend routine histopathologic analysis of gastric specimens after LSG in severely obese patients.
